# Pediatric near-drownings: clinical insights and prediction of life-threatening events

**DOI:** 10.3389/fped.2025.1700437

**Published:** 2026-01-12

**Authors:** Asmaa F. Sharif, Mohammed S. Alwarhi, Abdulaziz S. Alshamrani, Adel O. Alharbi, Nasser M. Alwayil, Turki N. Alharbi, Abdulwahab S. Alsarhan, Abdulrhman S. Alghamdi

**Affiliations:** 1 Department of Clinical Medical Sciences, College of Medicine, Dar Al-Uloom University, Riyadh, Saudi Arabia; 2 Forensic Medicine and Clinical Toxicology Department, Faculty of Medicine, Tanta University, Gharbia Governorate, Egypt; 3 Department of Emergency Medicine, Emergency Medical Services, King Abdulaziz Medical City, Ministry of National Guard Health Affairs, Riyadh, Saudi Arabia; 4 Department of Respiratory Therapy, Security Forces Hospital, Riyadh, Saudi Arabia; 5 Department of Respiratory Therapy, King Abdulaziz Medical City, Ministry of National Guard Health Affairs, Riyadh, Saudi Arabia; 6 Department of Cardiovascular Technology, King Saud bin Abdulaziz University for Health Sciences, Riyadh, Saudi Arabia; 7 Emergency Medical Services Department, College of Applied Medical Sciences, King Saud Bin Abdulaziz University for Health Sciences, Riyadh, Saudi Arabia; 8 King Abdullah International Medical Research Center, Riyadh, Saudi Arabia; 9 Emergency Department, King Abdulaziz Medical City, Ministry of National Guard Health Affairs, Riyadh, Saudi Arabia

**Keywords:** drowning, hypoxic ischemic encephalopathy, mechanical ventilation, mortality, pediatrics, severe outcomes

## Abstract

**Introduction:**

Drowning is a leading cause of mortality in children worldwide. Near-drowning is a state of survival after asphyxia due to submersion in water. Drowning-related incidents have significantly increased in developed countries. Even survivors suffer from various long-lasting morbidities. Early prediction of mortality and morbidly life-threatening events is life-saving and guides decision-making in such critical situations. This study aimed to identify early predictors of mortality and other unfavorable outcomes (hypoxic brain injury and respiratory failure necessitating mechanical ventilation) in children diagnosed with near-drowning.

**Methods:**

This retrospective cross-sectional study enrolled 247 children who nearly drowned and were admitted to National Guard Hospitals, Saudi Arabia, between 2,015 and 2024. The studied patients were categorized into two groups: patients with favorable outcomes and those with unfavorable outcomes. The all-subset regression method was employed to identify the best predictors for the investigated outcomes.

**Results:**

The death rate and incidence of brain insults constituted 15% each, while 25% of the investigated patients were complicated by respiratory failure. Most admissions (60%) occurred in the summer, and 86% of drownings occurred at swimming pools. Death was significantly associated with greater prehospital delay (time from exposure until arrival at emergency services) (*p* < 0.05). A proposed model that exhibited a performance of 92% identified the Glasgow Coma Scale score on admission as a significant predictor of brain insults. Significant predictors of mortality included lower bicarbonate levels [adjusted odds ratio (OR) = 0.598, *p* < 0.001], higher sodium levels (adjusted OR = 1.802, *p* < 0.001), lower chloride levels (adjusted OR = 0.721, *p* = 0.008), and greater red cell distribution width (adjusted OR = 2.158, *p* = 0.009).

**Conclusion:**

This study identified several available laboratory markers as early predictors of mortality in pediatric near-drowning patients. These markers help to identify patients at increased risk of life-threatening events when combined with established clinical predictors. This study emphasized the critical roles of prehospital factors, initial clinical presentation, and physiological derangements in pediatric near-drowning patients. The observed findings highlight possible areas accessible for public health interventions, including intensifying preventive measures at night, in the summer, and in swimming pools. Offering optimum on-scene resuscitation and rapid hospital transfer are other recommended actions expected to be associated with fewer adverse events.

## 1 Introduction

Drowning is a global issue that affects pediatric health and well-being. Recent studies have identified it as one of the leading causes of morbidity and mortality in children worldwide (1). Moreover, drowning has become the third leading cause of unintentional death among children (2). There are geographical differences in drowning rates, with low- and middle-income countries accounting for more than 90% of the total, showing rates up to 3.4 times higher than those in high-income countries (2). Even surviving children may face various neurological complications due to drowning (3).

The World Health Organization (WHO) has attempted to prevent drowning and find effective strategies to reduce deaths and injuries in all aquatic environments globally. These efforts aim to implement measures such as barriers to controlling water access and establishing day care centers for preschool children to avert drowning. Furthermore, the WHO has invested in research investigating critical questions concerning drowning prevention (4). Despite global efforts, the number of drowning-related incidents has significantly increased in developed countries (5). Many drowning cases present with cardiac arrest (6). Although drowning accounts for only 3% of emergency admissions, it is significantly associated with increased rates of morbid life-threatening events and mortality compared with trauma-related admissions (7). Numerous definitions of “drowning” exist. The most standardized definition of drowning is “*experiencing respiratory impairment from submersion or immersion in liquid*” (4). Near-drowning is a state of survival, at least temporarily, after asphyxia due to submersion in water (8). Immersion of the entire body or head leads to temporary suppression of the respiratory stimulus, which lasts for a maximum of a few minutes, after which involuntary water aspiration may occur, triggering reflex laryngospasm. With increasing hypercapnia and hypoxia, consciousness, as well as protective reflexes, is lost, which exacerbates further aspiration of large amounts of water and stomach contents (9). Pulmonary edema and acute respiratory distress rapidly ensue, followed by bradycardia and circulatory arrest (10).

The duration of submersion, the need for cardiopulmonary resuscitation (CPR), and its duration, as well as the return of spontaneous circulation, are considered primary indicators of drowning outcomes (3). Additionally, the risk of drowning in children may be influenced by environmental factors and inadequate public health funding (11). Children living in low- and middle-income countries die significantly more often than do those living in high-income countries. Child drowning fatalities are particularly elevated among the poorest and least educated populations, especially in rural areas near bodies of water, where resources to mitigate water-related risks are limited. Factors such as maternal illiteracy, seasonality, and lower socioeconomic status contribute to a heightened risk of drowning incidents (12). Inadequate supervision of children and a lack of swimming ability are major factors leading to these tragedies. Lacking safety education and first aid training among caregivers, who are frequently preoccupied with other tasks, is another reported risk factor for increased drowning in children (13).

To emphasize the importance of this issue, epidemiological studies have suggested several preventive strategies that have been found to be effective in reducing drowning incidents, such as adult supervision, early swimming lessons, pool fencing, and pool covers (14). Early prediction of mortality and morbidly life-threatening events, including neurological insults and cardiorespiratory arrest, is life-saving and guides decision-making in such critical situations (15). Early stratification of the victims admitted according to the risk of death and one or more severe outcomes seems vital. Given the limited research on pediatric near-drowning, particularly regarding exposure circumstances and initial clinical presentations, this study aims to identify early predictors of mortality and other life-threatening outcomes, such as hypoxic encephalopathy and respiratory failure requiring mechanical ventilation (MV), in pediatric patients who are diagnosed with near-drowning. The findings obtained could inform decision-making for emergency physicians and support targeted training programs and educational campaigns.

## 2 Subjects and methods

### 2.1 Study design and setting

This retrospective cross-sectional study utilized medical records of patients under 18 years of age who were diagnosed with near-drowning/related conditions and admitted to National Guard Hospitals, Saudi Arabia, between 2015 and 2024. Initial diagnoses were based on the International Classification of Diseases (ICD-10), specifically code T75.1XXS, which pertains to unspecified effects of drowning and nonfatal submersion, i.e., sequelae (16). Confirmation of the near-drowning diagnosis was made by the attending physician's documentation in medical records. The recruited cases were retrieved from medical records using the specified ICD codes.

### 2.2 Sampling and sample size calculation

Nonrandomized convenience sampling was adapted to approach all eligible patients. This approach was necessitated by the retrospective nature of the study, where we included all consecutive, eligible patients from the medical records during the specified period. However, to ensure that the study investigated the planned outcomes, the sample size was calculated via OpenEpi software (version 3) (17). Considering the death rate of 13.1% (7) and hypoxic ischemic encephalopathy rate of 13.8% (18) among admitted pediatric patients diagnosed with near-drowning, in an infinite population and a 95% confidence interval, the minimum sample sizes should be 175 and 183 patients, respectively. We obtained 247 patients.

### 2.3 Inclusion and exclusion criteria

The present study was conducted on admitted pediatric patients (younger than 18 years) who were diagnosed with near-drowning and who presented at the National Guards Hospital during the study period (2015–2024). All pediatric patients with complete medical records were considered eligible regardless of the manner and circumstances of the drowning. Children identified as dead before hospital admission, as well as those with missing or incomplete medical records, were excluded. Patients with preexisting neurological or developmental disabilities not related to the drowning incident and patients with an unconfirmed diagnosis of near drowning were excluded.

### 2.4 Data collection tool

Data from the investigated patients were accessed through the BESTCare system (bestcare.ezcaretech.com). BESTcare is a robust health information system that uses electronic medical records to store and manage patients' data from birth. For every included patient, we reported the patient's characteristics, including demographic data and circumstances of drowning: age, sex, history of chronic disease, timing and month of drowning, and the drowning medium if known (swimming pool, natural water, bathtub, water tank, bucket, and others). Additionally, we reported the delay time from exposure until arrival of the emergency services and the mode of transport to the hospital (ambulance, privately owned vehicle, helicopter, and others). Receiving cardiopulmonary resuscitation (CPR) was mentioned.

### 2.5 Definition of variables

The patient's condition on admission was reported, including Glasgow Coma Scale (GCS) scores ranging from 3 to 15. We reported the main presenting complaints (dyspnea, throat and nasal secretions, loss of consciousness, and cardiac arrest) and any signs of external body trauma. Positive findings in general and specific body systems on examination, as well as vital data on admission, including systolic and diastolic blood pressures (mmHg), pulse (beat/min), respiratory rate (cycle/min), temperature in Celsius, and oxygen saturation%, were documented.

The results of the conducted laboratory investigations are reported below.

- Arterial blood gas analysis [pH, PaCO_2_ (mmHg), and HCO_3_ (mEq/L)].
- Electrolytes [Na (mmol/L), K (mmol/L), and Cl (mmol/L)]
- Blood glucose level (mmol/L), serum urea level (mmol/L), total bilirubin level (*μ*mol/L), and creatinine level (μmol/L)
- The complete blood count (CBC) included the red blood cell count (x10^12^/L), white blood cell and differential leucocyte counts (x10^9^/L), hematocrit (%), mean corpuscular volume (MCV) in fL, mean corpuscular hemoglobin (MCH) in pg, mean corpuscular hemoglobin concentration (MCHC) in g/dL, platelet count (x10^9^/L), and percentage of red cell distribution width (RDW).

The findings obtained from the radiological studies, including chest radiography and brain imaging, were analyzed. The patients who received treatment, including vasopressor therapy, sedation, antibiotic use, and intensive care unit (ICU) utilization, were also described. The length of stay (LOS) in the hospital, in hours, from admission until discharge was reported.

### 2.6 Outcomes and grouping

The studied patients were categorized according to the occurrence of life-threatening events into two groups: patients with favorable outcomes and those with severe unfavorable outcomes. Unfavorable outcomes were defined as a composite of one or more of the following: endpoint of death, respiratory failure proven by the need for MV, and hypoxic ischemic brain insults (7). Acute respiratory failure was confirmed by retrieving the relevant ICD-10 code J96.0 from the medical records, which described “*a state of acute respiratory failure, unspecified whether with hypoxia or hypercapnia*”*.* Likewise, the diagnosis of brain insult was established if the medical record mentioned the code G93.1, which describes “*all causes of anoxic/ischemic/hypoxic brain injury, not elsewhere* classified” (16).

### 2.7 Compliance with ethical standards

The current study commenced after approval was obtained from the King Abdullah International Medical Research Center (KAIMRC) Institutional Review Board (IRB Approval number NRR24/066/10). This study adheres to the Declaration of Helsinki and its later amendments. Medical records were handled anonymously, and patients' confidentiality was preserved via a coding system for every medical report. Owing to the retrospective observational nature of the study, the IRB waived the requirement of informed consent.

### 2.8 Data analysis

Analyses were conducted via the R statistical language (version 4.5.0) (19). The normality of the distribution was tested via the Shapiro‒Wilk test and with Q‒Q plots. Continuous numerical variables following a normal distribution were summarized using the mean, standard deviation (SD), and range (minimum to maximum values). For nonnormally distributed numerical variables, the median and interquartile range (IQR, ^25^th^–75^th percentiles) were used. Comparisons for nonnormally distributed variables were performed via the Wilcoxon rank sum test. Comparisons of variables that may be affected by age (vital signs and laboratory results) were performed via least-squares linear regression. Categorical variables are summarized as counts and frequencies. The associations were tested via Pearson's chi-square test for independence of observations (for nominal variables, replaced by Fisher's exact test if the expected count was less than 5 in 20% or more of the cells) or the chi-square test for trend in proportions (for ordinal variables). A *p* value <0.05 was selected to interpret the results of the statistical tests.

The all-subset regression method was employed to identify the best combination of predictors for the outcome variable. This approach systematically evaluates all possible combinations of candidate variables to determine the models that best balance goodness of fit and model simplicity, using the corrected Akaike information criterion (AIC). Comparing models across all subsets helps avoid overfitting and enhances the robustness of model selection, particularly when multiple predictors are considered. All continuous predictors were entered into the multivariable logistic regression models as continuous variables using their original units of measurement. The regression coefficients (*β*) therefore represent the change in log odds per 1-unit increase in each variable. Because pH varies within a narrow physiologic range, its adjusted odds ratio (OR) was reported per 0.01-unit increase for interpretability and was calculated from the original model coefficient as OR = exp (*β* × 0.01). All model fit statistics correspond to variables entered in their original scales.

To evaluate potential confounding by resuscitation or altered consciousness, we examined whether the laboratory and clinical predictors identified in the regression analyses were influenced by these confounders. Receiving CPR and loss of consciousness on admission were included as covariates in the final respective multivariate models for each outcome (hypoxic brain insult, respiratory failure necessitated MV, and mortality) to adjust for confounding by severity and initial resuscitation efforts. We compared the resulting regression coefficients with those from the original models to assess changes in effect estimates. Predictors whose coefficients changed <10% were considered independent, whereas larger changes indicated potential partial confounding. However, the mortality predictive model included the loss of consciousness variable, so we rerun that model after implementing Firth's bias-reduction method via the “brglmFit” method in the brglm2 package. In addition, the potential confounding or nonlinear effects of age on all the outcomes of interest were assessed by including age as a continuous covariate in all the models and by modeling via flexible smooth terms in generalized additive models (GAMs).

## 3 Results

This study involved 247 patients, with a median age of 2.3 years, of whom 58% were males and 42% were females. Unfavorable outcomes were observed in 31% of patients, including a 15% mortality rate. Notably, 25% of patients experienced respiratory failure, and 15% suffered from brain insults. Approximately 60% of nearly drowned children were admitted during the summer months (July–September). Notably, 62% of the near-drowning incidents occurred at night, and 86% of the incidents occurred in swimming pools. All investigated patients were admitted after unintentional exposure. These circumstances were consistent among all patients, regardless of their outcomes, as shown in Table 1. Table 2 indicates that emergency services commenced after a median delay of 18 min (0.3 h), with longer delays significantly associated with higher mortality. Approximately 69% of patients were transported by private cars, and 25% were transported by ambulances. Ambulance transport was associated with a greater incidence of unfavorable outcomes. Helicopter transport was particularly associated with severe outcomes; brain insults and respiratory failure were reported in 60% and 80% of helicopter-transported patients, respectively (*p* < 0.001).

**Table 1:** Demographics and history of exposure in the studied patients according to the investigated outcomes.

Characteristic^a^	All victims	Unfavorable outcomes		Brain insult		Respiratory failure and MV		Mortality	
	Overall *N* = 247 (100%)	No *N* = 170 (69%)	Yes *N* = 77 (31%)	No *N* = 211 (85%)	Yes *N* = 36 (15%)	No *N* = 186 (75%)	Yes *N* = 61 (25%)	No *N* = 210 (85%)	Yes *N* = 37 (15%)
Age Median [IQR] (Range)	2.3 [1.6–4.0] (0.1 −18.0)	2.1 [1.5 −4.0] (0.1–18.0)	2.7 [1.7–4.0] (1.0–10.0)	2.3 [1.6–4.0] (0.1–18.0)	2.3 [1.6–3.0] (1.0–10.0)	2.2 [1.5–4.0] (0.1–18.0)	2.6 [1.7–3.0] (1.0–10.0)	2.3 [1.6–4.0] (0.1 −18.0)	2.2 [1.5–4.0] (1.0–9.0)
*p* value^b^		0.216*c*		0.576*c*		0.473*c*		0.819*c*	
Sex, *n* (%)									
Female	103 (42)	70 (41/68)	33 (43/32)	87 (41/84)	16 (44/16)	76 (41/74)	27 (44/26)	88 (42/85)	15 (41/15)
Male	144 (58)	100 (59/69)	44 (57/31)	124 (59/86)	20 (56/14)	110 (59/76)	34 (56/24)	122 (58/85)	22 (59/15)
*p* value^b^		0.804*d*		0.718*d*		0.640*d*		0.877*d*	
History of chronic diseases, *n* (%)									
None	215 (87)	146 (86/68)	69 (90/32)	181 (86/84)	34 (94/16)	161 (87/75)	54 (89/25)	181 (86/84)	34 (92/16)
Bronchial asthma	13 (5)	10 (6/77)	3 (4/23)	12 (6/92)	1 (3/7.7)	10 (5/77)	3 (5/23)	11 (5/85)	2 (5/15)
Autism	6 (2)	3 (2/50)	3 (4/50)	5 (2/83)	1 (3/17)	4 (2/67)	2 (3/33)	5 (2/83)	1 (3/17)
Congenital syndrome	6 (2)	5 (3/83)	1 (1/17)	6 (3/100)	0 (0/0)	5 (3/83)	1 (2/17)	6 (3/100)	0 (0/0)
Epilepsy	4 (2)	4 (2/100)	0 (0/0)	4 (2/100)	0 (0/0)	4 (2/100)	0 (0/0)	4 (2/100)	0 (0/0)
Renal disorders	2 (1)	1 (1/50)	1 (1/50)	2 (1/100)	0 (0/0)	1 (1/50)	1 (2/50)	2 (1/100)	0 (0/0)
DM	1 (0)	1 (1/100)	0 (0/0)	1 (0/100)	0 (0/0)	1 (1/100)	0 (0/0)	1 (0/100)	0 (0/0)
*p* value^b^		0.644*e*		0.939*e*		0.864*e*		0.984*e*	
Month of admission, *n* (%)									
January	6 (2)	4 (2/67)	2 (3/33)	4 (2/67)	2 (6/33)	4 (2/67)	2 (3/33)	5 (2/83)	1 (3/17)
February	8 (3)	5 (3/63)	3 (4/38)	8 (4/100)	0 (0/0)	5 (3/63)	3 (5/38)	7 (3/88)	1 (3/13)
March	14 (6)	12 (7/86)	2 (3/14)	13 (6/93)	1 (3/7.1)	13 (7/93)	1 (2/7.1)	14 (7/100)	0 (0/0)
April	15 (6)	10 (6/67)	5 (6/33)	12 (6/80)	3 (8/20)	10 (5/67)	5 (8/33)	11 (5/73)	4 (11/27)
May	23 (9)	17 (10/74)	6 (8/26)	20 (9/87)	3 (8/13)	20 (11/87)	3 (5/13)	21 (10/91)	2 (5/8.7)
June	34 (14)	23 (14/68)	11 (14/32)	29 (14/85)	5 (14/15)	25 (13/74)	9 (15/26)	27 (13/79)	7 (19/21)
July	50 (20)	34 (20/68)	16 (21/32)	45 (21/90)	5 (14/10)	37 (20/74)	13 (21/26)	44 (21/88)	6 (16/12)
August	33 (13)	24 (14/73)	9 (12/27)	29 (14/88)	4 (11/12)	25 (13/76)	8 (13/24)	29 (14/88)	4 (11/12)
September	32 (13)	20 (12/63)	12 (16/38)	25 (12/78)	7 (19/22)	25 (13/78)	7 (11/22)	24 (11/75)	8 (22/25)
October	14 (6)	9 (5/64)	5 (6/36)	11 (5/79)	3 (8/21)	9 (5/64)	5 (8/36)	13 (6/93)	1 (3/7.1)
November	9 (4)	6 (4/67)	3 (4/33)	8 (4/89)	1 (3/11)	7 (4/78)	2 (3/22)	6 (3/67)	3 (8/33)
December	9 (4)	6 (4/67)	3 (4/33)	7 (3/78)	2 (6/22)	6 (3/67)	3 (5/33)	9 (4/100)	0 (0/0)
*p* value^b^		0.458*f*		0.455*f*		0.632*f*		0.642*f*	
Time of drowning, *n* (%)									
Daytime	82 (33)	60 (35/73)	22 (29/27)	72 (34/88)	10 (28/12)	63 (34/77)	19 (31/23)	74 (35/90)	8 (22/9.8)
Night	152 (62)	103 (61/68)	49 (64/32)	131 (62/86)	21 (58/14)	115 (62/76)	37 (61/24)	126 (60/83)	26 (70/17)
Undetermined	13 (5)	/7 (4/54)	6 (8/46)	8 (4/62)	5 (14/38)	8 (4/62)	5 (8/38)	10 (5/77)	3 (8/23)
*p* value^b^		0.325*e*		0.063*e*		0.484*e*		0.189*e*	
Drowning medium, *n* (%)									
Swimming pool	212 (86)	144 (85/68)	68 (88/32)	180 (85/85)	32 (89/15)	157 (84/74)	55 (90/26)	179 (85/84)	33 (89/16)
Bathtub	9 (4)	7 (4/78)	2 (3/22)	8 (4/89)	1 (3/11)	7 (4/78)	2 (3/22)	9 (4/100)	0 (0/0)
Natural water	8 (3)	6 (4/75)	2 (3/25)	7 (3/88)	1 (3/13)	6 (3/75)	2 (3/25)	6 (3/75)	2 (5/25)
Water tank	6 (2)	3 (2/50)	3 (4/50)	5 (2/83)	1 (3/17)	4 (2/67)	2 (3/33)	5 (2/83)	1 (3/17)
Bucket	2 (1)	2 (1/100)	0 (0/0)	2 (1/100)	0 (0/0)	2 (1/100)	0 (0/0)	2 (1/100)	0 (0/0)
Others	10 (4)	8 (5/80)	2 (3/20)	9 (4/90)	1 (3/10)	10 (5/100)	0 (0/0)	9 (4/90)	1 (3/10)
*p* value^b^		0.805*e*		>0.999*e*		0.468*e*		0.751*e*	

Percents are expressed as column/row percentages: significantly lower probability than expected under the null hypothesis when adjusted residuals are examined with the Bonferroni correction $+: significantly higher probability than expected under the null hypothesis when adjusted residuals are examined with the Bonferroni correction.

^a^ IQR, Interquartile range (25th–75th percentiles); n, number.

^b^ * Significant at *p* < 0.05, *c* Wilcoxon rank sum test, *d* Pearson's chi-square test, *e* Fisher's exact test, *f* Chi-square test for trend in proportion.

**Table 2:** Circumstances of drowning and clinical findings on admission in the studied patients according to the investigated outcomes.

Characteristic a	All victims	Unfavorable outcomes		Brain insult		Respiratory failure and MV		Mortality	
	Overall *N* = 247 (100%)	No *N* = 170 (69%)	Yes *N* = 77 (31%)	No *N* = 211 (85%)	Yes *N* = 36 (15%)	No *N* = 186 (75%)	Yes *N* = 61 (25%)	No *N* = 210 (85%)	Yes *N* = 37 (15%)
Delay time (h), Median [IQR] (Range)	0.3 [0.3–1.0] (0.2–48.0)	0.3 [0.3–1.0] (0.3–48.0)	0.5 [0.3–0.7] (0.2–45.0)	0.3 [0.3–1.0] (0.2–48.0)	0.5 [0.3–0.7] (0.3–40.0)	0.5 [0.3–1.0] (0.3–48.0)	0.3 [0.3–0.5] (0.2–45.0)	0.3 [0.3–1.0] (0.3–48.0)	0.5 [0.3–1.0] (0.2–45.0)
*p* value^b^		0.784^c^		0.661^c^		0.314^c^		0.038*^c^	
Transportation mode, *n* (%)									
Private car	170 (69)	142 (84/84) $+	28 (36/16) $-	158 (75/93) $+	12 (33/7.1) $-	152 (82/89) $+	18 (30/11) $-	155 (74/91) $+	15 (41/8.8) $-
Ambulance	62 (25)	19 (11/31) $-	43 (56/69) $+	43 (20/69) $-	19 (53/31) $+	24 (13/39) $-	38 (62/61) $+	42 (20/68) $-	20 (54/32) $+
Helicopter	5 (2)	1 (1/20)	4 (5/80)	2 (1/40) $-	3 (8/60) $+	1 (1/20) $-	4 (7/80) $+	3 (1/60)	2 (5/40)
Others	10 (4)	8 (5/80)	2 (3/20)	8 (4/80)	2 (6/20)	9 (5/90)	1 (2/10)	10 (5/100)	0 (0/0)
*p* value^b^		<0.001*^d^		<0.001*^d^		<0.001*^d^		<0.001*^d^	
Immediate CPR after discovery, *n* (%)									
No	122 (49)	109 (64/89)	13 (17/11)	116 (55/95)	6 (17/4.9)	114 (61/93)	8 (13/6.6)	116 (55/95)	6 (16/4.9)
Yes	125 (51)	61 (36/49)	64 (83/51)	95 (45/76)	30 (83/24)	72 (39/58)	53 (87/42)	94 (45/75)	31 (84/25)
*p* value^b^		<0.001*^e^		<0.001*^e^		<0.001*^e^		<0.001*^e^	
Loss of consciousness before admission, *n* (%)	160 (65)	89 (52/56)	71 (92/44)	126 (60/79)	34 (94/21)	103 (55/64)	57 (93/36)	125 (60/78)	35 (95/22)
*p* value^b^		<0.001*^e^		<0.001*^e^		<0.001*^e^		<0.001*^e^	
GCS on admission, Mea^n^ ± SD (Range)	12 ± 5 (3–15)	15 ± 1 (8–15)	5 ± 3 (3–15)	13 ± 4 (3–15)	4 ± 2 (3–11)	14 ± 3 (3–15)	6 ± 4 (3–15)	13 ± 3 (3–15)	3 ± 0 (3–4)
*p* value^b^		<0.001*^c^		<0.001*^c^		<0.001*^c^		<0.001*^c^	
Cardiac arrest on admission, *n* (%)	11 (4)	0 (0/0)	11 (14/100)	6 (3/55)	5 (14/45)	4 (2/36)	7 (11/64)	2 (1/18)	9 (24/82)
*p* value^b^		<0.001*^d^		0.012*^d^		0.006*^d^		<0.001*^d^	
Dyspnea and SOB, *n* (%)	8 (3)	6 (4/75)	2 (3/25)	8 (4/100)	0 (0/0)	6 (3/75)	2 (3/25)	8 (4/100)	0 (0/0)
*p* value^b^		>0.999^d^		0.607^d^		>0.999^d^		0.610^d^	
Throat & nose secretions, *n* (%)	53 (21)	21 (12/40)	32 (42/60)	42 (20/79)	11 (31/21)	29 (16/55)	24 (39/45)	38 (18/72)	15 (41/28)
*p* value^b^		<0.001*^e^		0.150^e^		<0.001*^e^		0.002*^e^	
Pupil, *n* (%)									
Normal	190 (77)	167 (98/88) $+	23 (30/12) $-	183 (87/96) $+	7 (19/3.7) $-	170 (91/89) $+	20 (33/11) $-	188 (90/99) $+	2 (5/1.1) $-
Mydriasis	34 (14)	0 (0/0) $-	34 (44/100) $+	15 (7/44) $-	19 (53/56) $+	11 (6/32) $-	23 (38/68) $+	7 (3/21) $-	27 (73/79) $+
Miosis	16 (6)	2 (1/13) $-	14 (18/88) $+	9 (4/56) $-	7 (19/44) $+	3 (2/19) $-	13 (21/81) $+	13 (6/81)	3 (8/19)
Fixed dilated	7 (3)	1 (1/14) $-	6 (8/86) $+	4 (2/57)	3 (8/43)	2 (1/29) $-	5 (8/71) $+	2 (1/29) $-	5 (14/71) $+
*p* value^b^		<0.001*^d^		<0.001*^d^		<0.001*^d^		<0.001*^d^	
Crepitations, *n* (%)	28 (11)	9 (5/32)	19 (25/68)	20 (9/71)	8 (22/29)	10 (5/36)	18 (30/64)	21 (10/75)	7 (19/25)
*p* value^b^		<0.001*^e^		0.042*^d^		<0.001*^e^		0.154^d^	
Wheezy chest, *n* (%)	12 (5)	5 (3/42)	7 (9/58)	9 (4/75)	3 (8/25)	5 (3/42)	7 (11/58)	10 (5/83)	2 (5/17)
*p* value^b^		0.053^d^		0.391^d^		0.011*^d^		0.697^d^	
Respiratory Distress grade, *n* (%)									
0	151 (61)	130 (76/86)	21 (27/14)	139 (66/92)	12 (33/7.9)	138 (74/91)	13 (21/8.6)	139 (66/92)	12 (32/7.9)
1	30 (12)	28 (16/93)	2 (3/6.7)	29 (14/97)	1 (3/3.3)	28 (15/93)	2 (3/6.7)	29 (14/97)	1 (3/3.3)
2	15 (6)	11 (6/73)	4 (5/27)	15 (7/100)	0 (0/0)	11 (6/73)	4 (7/27)	15 (7/100)	0 (0/0)
3	13 (5)	1 (1/7.7)	12 (16/92)	8 (4/62)	5 (14/38)	2 (1/15)	11 (18/85)	13 (6/100)	0 (0/0)
4	38 (15)	0 (0/0)	38 (49/100)	20 (9/53)	18 (50/47)	7 (4/18)	31 (51/82)	14 (7/37)	24 (65/63)
*p* value^b^		<0.001*^f^		<0.001*^f^		<0.001*^f^		<0.001*^f^	
Abdominal tenderness & rigidity, *n* (%)	21 (9)	9 (5/43)	12 (16/57)	15 (7/71)	6 (17/29)	12 (6/57)	9 (15/43)	11 (5/52)	10 (27/48)
*p* value^b^		0.007*^e^		0.096^d^		0.044*^e^		<0.001*^d^	
Seizures, *n* (%)	19 (8)	3 (2/16)	16 (21/84)	7 (3/37)	12 (33/63)	5 (3/26)	14 (23/74)	15 (7/79)	4 (11/21)
*p* value^b^		<0.001*^e^		<0.001*^d^		<0.001*^d^		0.500^d^	
Arrest during admitted, *n* (%)	95 (38)	28 (16/29)	67 (87/71)	61 (29/64)	34 (94/36)	43 (23/45)	52 (85/55)	58 (28/61)	37 (100/39)
*p* value^b^		<0.001*^e^		<0.001*^e^		<0.001*^e^		<0.001*^e^	
External body trauma, *n* (%)	11 (4)	8 (5/73)	3 (4/27)	11 (5/100)	0 (0/0)	8 (4/73)	3 (5/27)	11 (5/100)	0 (0/0)
*p* value^b^		>0.999^d^		0.375^d^		0.735^d^		0.378^d^	

Percents are expressed as column/row percentages; $-: significantly lower probability than expected under the null hypothesis for examining adjusted residuals with Bonferroni correction$+: significantly higher probability than expected under the null hypothesis for examining adjusted residuals with Bonferroni correction.

^a^ IQR: Interquartile range (25th–75th percentiles); n, number.

^b^ * Significant at *p* < 0.05.

^c^ Wilcoxon rank sum test.

^d^ Fisher's exact test

^e^ Pearson's chi-square test.

^f^ Chi-square test for trend in proportions.

As shown in Table 3, mortality was strongly associated with lower blood pressure and pulse. Moreover, low systolic blood pressure and increased respiratory rate were correlated with an increased likelihood of brain insults (*p* < 0.05). A lower body temperature was significantly linked to all adverse outcomes, whereas oxygen saturation was notably reduced in patients with respiratory failure and death (*p* < 0.001). Additionally, reduced pH and bicarbonate levels and increased PaCO_2_ were significantly associated with the development of unfavorable outcomes (*p* < 0.001). Serum sodium and potassium levels decrease in patients facing adverse outcomes, whereas blood glucose and serum creatinine levels increase. Patients with unfavorable outcomes had higher MCVs but lower MCHCs (*p* < 0.05). Table 4 shows that all patients with computerized tomography (CT) abnormalities experienced one or more unfavorable outcomes and received significantly higher doses of vasopressors, sedations, and antibiotics (*p* < 0.001).

**Table 3:** Vital signs and laboratory findings in the studied patients according to the investigated outcomes.

Characteristic^a^	All victims	Unfavorable outcomes		Brain insult		Mechanical ventilation		Mortality	
	Overall *N* = 247 (100%)	No *N* = 170 (69%)	Yes *N* = 77 (31%)	No *N* = 211 (85%)	Yes *N* = 36 (15%)	No *N* = 186 (75%)	Yes *N* = 61 (25%)	No *N* = 210 (85%)	Yes *N* = 37 (15%)
I. Vital signs on admission									
Systolic Blood Pressure (mmHg), Mean ± SD (Range)	105.5 ± 18.1 (41.0–170.0)	106.5 ± 13.6 (73.0–140.0)	102.6 ± 27.2 (41.0–170.0)	106.7 ± 15.9 (60.0–170.0)	98.2 ± 27.0 (41.0–155.0)	106.6 ± 13.5 (73.0–140.0)	102.2 ± 27.8 (41.0–170.0)	107.1 ± 16.4 (41.0–170.0)	86.8 ± 25.8 (59.0–155.0)
*p* value^b^		0.211^c^		0.025*^c^		0.155^c^		<0.001*^c^	
Diastolic Blood Pressure (mmHg), Mean ± SD (Range)	65.8 ± 16.8 (15.0–143.0)	66.9 ± 13.3 (40.0–114.0)	62.8 ± 23.9 (15.0–143.0)	66.7 ± 14.1 (22.0–114.0)	61.0 ± 27.1 (15.0–143.0)	67.1 ± 13.2 (40.0–114.0)	62.1 ± 24.4 (15.0–143.0)	67.2 ± 15.0 (15.0–143.0)	51.3 ± 26.2 (21.0–103.0)
*p* value^b^		0.148^c^		0.123^c^		0.079^c^		<0.001*^c^	
Pulse (beat/min), Mean ± SD (Range)	124.3 ± 25.4 (40.0–199.0)	124.7 ± 22.8 (41.0–199.0)	123.4 ± 32.1 (40.0–185.0)	124.1 ± 23.7 (40.0–199.0)	125.7 ± 34.5 (54.0–185.0)	125.0 ± 23.3 (41.0–199.0)	122.3 ± 31.2 (40.0–185.0)	125.5 ± 23.7 (41.0–199.0)	112.6 ± 38.4 (40.0–185.0)
*p* value^b^		0.590^c^		0.996^c^		0.371^c^		0.007*^c^	
Respiratory rate (cycle/min), Mean ± SD (Range)	30.9 ± 7.0 (14.0–62.0)	30.3 ± 6.4 (19.0–62.0)	32.9 ± 8.2 (14.0–56.0)	30.5 ± 6.6 (14.0–62.0)	34.0 ± 8.4 (20.0–56.0)	30.5 ± 6.7 (19.0–62.0)	32.2 ± 7.7 (14.0–50.0)	30.7 ± 6.8 (19.0–62.0)	33.3 ± 8.7 (14.0–50.0)
*p* value^b^		0.026*^c^		0.024*^c^		0.182^c^		0.412^c^	
Temperature (Celsius), Mean ± SD (Range)	36.2 ± 1.4 (28.0–39.6)	36.6 ± 0.8 (34.0–39.6)	35.1 ± 2.1 (28.0–39.5)	36.3 ± 1.2 (31.0–39.6)	35.3 ± 2.1 (28.0–39.5)	36.5 ± 1.1 (28.0–39.6)	35.2 ± 1.9 (31.0–39.5)	36.5 ± 1.1 (31.2–39.6)	34.2 ± 2.1 (28.0–36.9)
*p* value^b^		<0.001*^c^		<0.001*^c^		<0.001*^c^		<0.001*^c^	
O_2_ saturation%, Mean ± SD (Range)	96.4 ± 7.4 (36.9–100.0)	97.4 ± 5.3 (36.9–100.0)	93.6 ± 11.1 (44.0–100.0)	96.7 ± 7.3 (36.9–100.0)	94.6 ± 7.8 (69.0–100.0)	97.4 ± 5.3 (36.9–100.0)	93.3 ± 11.3 (44.0–100.0)	97.0 ± 6.3 (36.9–100.0)	90.2 ± 13.5 (50.0–100.0)
*p* value^b^		<0.001*^c^		0.171^c^		<0.001*^c^		<0.001*^c^	
II. Laboratory investigations									
pH, Mean ± SD (Range)	7.24 ± 0.20 (6.50–7.48)	7.32 ± 0.06 (7.10–7.48)	7.09 ± 0.27 (6.50–7.47)	7.28 ± 0.15 (6.50–7.48)	7.10 ± 0.30 (6.59–7.47)	7.31 ± 0.11 (6.50–7.48)	7.10 ± 0.26 (6.50–7.47)	7.29 ± 0.12 (6.70–7.48)	6.90 ± 0.31 (6.50–7.47)
*p* value^b^		<0.001*^c^		<0.001*^c^		<0.001*^c^		<0.001*^c^	
HCO₃ (mEq/L), Mean ± SD (Range)	20.0 ± 4.2 (5.7–29.5)	21.0 ± 2.2 (14.2–28.4)	18.1 ± 6.1 (5.7–29.5)	20.5 ± 3.1 (8.6–28.4)	17.9 ± 7.0 (5.7–29.5)	21.0 ± 2.3 (14.2–28.4)	18.0 ± 6.3 (5.7–29.5)	20.7 ± 3.0 (9.8–29.5)	15.1 ± 7.3 (5.7–27.5)
*p* value^b^		<0.001*^c^		0.005*^c^		<0.001*^c^		<0.001*^c^	
PaCO_2_ (mmHg), Mean ± SD (Range)	46.6 ± 16.1 (29.9–135.5)	41.1 ± 6.7 (30.0–70.2)	56.9 ± 22.5 (29.9–135.5)	44.1 ± 12.1 (29.9–98.1)	56.8 ± 24.9 (32.0–135.5)	41.1 ± 6.6 (30.0–70.2)	57.7 ± 22.8 (29.9–135.5)	44.3 ± 13.3 (29.9–122.6)	63.8 ± 24.0 (36.0–135.5)
*p* value^b^		<0.001*^c^		<0.001*^c^		<0.001*^c^		<0.001*^c^	
Na (mmol/L), Mean ± SD (Range)	137.3 ± 4.9 (124.3–168.5)	136.8 ± 3.4 (128.0–146.0)	138.6 ± 7.3 (124.3–168.5)	136.9 ± 3.7 (128.0–152.0)	139.4 ± 8.5 (124.3–168.5)	136.7 ± 3.4 (128.0–146.0)	138.9 ± 7.3 (124.3–168.5)	136.7 ± 3.7 (124.3–146.0)	142.6 ± 9.6 (125.0–168.5)
*p* value^b^		0.024*^c^		0.010*^c^		0.004*^c^		<0.001*^c^	
K (mmol/L), Mean ± SD (Range)	3.96 ± 0.70 (2.00–7.22)	4.09 ± 0.60 (2.90–7.22)	3.66 ± 0.81 (2.00–6.11)	4.01 ± 0.64 (2.00–7.22)	3.72 ± 0.89 (2.47–6.11)	4.09 ± 0.60 (2.90–7.22)	3.64 ± 0.82 (2.00–6.11)	4.01 ± 0.65 (2.00–7.22)	3.49 ± 0.96 (2.47–6.11)
*p* value^b^		<0.001*^c^		0.027*^c^		<0.001*^c^		0.001*^c^	
Cl (mmol/L), Mean ± SD (Range)	106.5 ± 5.6 (96.0–141.0)	105.3 ± 2.7 (98.6–112.0)	109.4 ± 9.1 (96.0–141.0)	105.8 ± 3.7 (98.6–127.0)	110.4 ± 11.4 (96.0–141.0)	105.3 ± 2.7 (98.6–112.0)	109.7 ± 9.3 (96.0–141.0)	105.8 ± 3.5 (98.6–122.0)	112.8 ± 13.8 (96.0–141.0)
*p* value^b^		<0.001*^c^		0.002*^c^		<0.001*^c^		<0.001*^c^	
Blood glucose (mmol/L), Median [IQR] (Range)	6.3 [5.2–9.9] (3.0–33.4)	5.7 [4.9–7.4] (3.0–14.8)	11.1 [6.5–16.7] (3.9–33.4)	6.1 [5.0–8.4] (3.0–22.8)	13.2 [6.3–17.7] (4.6–33.4)	5.9 [5.0–7.5] (3.0–15.0)	11.1 [6.9–17.1] (4.3–33.4)	6.1 [5.1–8.4] (3.0–26.6)	13.5 [7.3–18.5] (3.9–33.4)
*p* value^b^		<0.001*^c^		<0.001*^c^		<0.001*^c^		<0.001*^c^	
Serum urea (mmol/L), Mean ± SD (Range)	4.0 ± 1.6 (0.8–9.5)	3.9 ± 1.3 (1.1–7.9)	4.2 ± 2.1 (0.8–9.5)	3.9 ± 1.4 (0.8–8.1)	4.4 ± 2.3 (1.2–9.5)	3.8 ± 1.3 (1.1–7.9)	4.2 ± 2.1 (0.8–9.5)	3.9 ± 1.4 (1.0–8.1)	4.7 ± 2.4 (0.8–9.5)
*p* value^b^		0.249^c^		0.164^c^		0.183^c^		0.044*^c^	
Serum creatinine (umol/L), Mean ± SD (Range)	45.9 ± 11.3 (25.0–96.0)	44.1 ± 8.5 (30.0–90.0)	49.6 ± 15.1 (25.0–96.0)	44.8 ± 9.4 (27.0–90.0)	50.6 ± 16.8 (25.0–96.0)	44.0 ± 8.4 (30.0–90.0)	50.1 ± 15.3 (25.0–96.0)	44.5 ± 9.6 (27.0–90.0)	57.0 ± 17.0 (25.0–96.0)
*p* value^b^		<0.001*^c^		0.001*^c^		<0.001*^c^		<0.001*^c^	
Total bilirubin (umol/L), Median [IQR] (Range)	4.8 [3.5–7.4] (1.5–23.6)	5.1 [4.0–8.2] (1.5–13.1)	4.4 [2.9–7.4] (1.5–23.6)	5.1 [3.8–8.1] (1.5–23.6)	4.5 [2.8–6.3] (1.5–13.8)	5.1 [4.0–8.2] (1.5–13.8)	4.4 [2.9–7.4] (1.5–23.6)	4.9 [3.8–7.4] (1.5–23.6)	3.7 [1.8–7.7] (1.5–9.7)
*p* value^b^		0.844^c^		0.275^c^		0.525^c^		0.536^c^	
RBCs (×10^12^/L), Mean ± SD (Range)	4.6 ± 0.5 (3.0–6.2)	4.7 ± 0.4 (3.0–6.2)	4.5 ± 0.5 (3.2–5.7)	4.7 ± 0.4 (3.0–6.2)	4.4 ± 0.5 (3.2–5.7)	4.7 ± 0.4 (3.0–6.2)	4.5 ± 0.5 (3.5–5.7)	4.7 ± 0.5 (3.0–6.2)	4.5 ± 0.5 (3.8–5.7)
*p* value^b^		0.004*^c^		0.012*^c^		0.027*^c^		0.113^c^	
WBCs (×10^9^/L), Mean ± SD (Range)	12.0 ± 5.2 (2.3–35.0)	11.4 ± 4.4 (3.0–28.2)	13.2 ± 6.3 (2.3–35.0)	11.9 ± 5.0 (2.5–35.0)	12.2 ± 5.7 (2.3–28.3)	11.5 ± 4.5 (3.0–28.2)	12.9 ± 6.4 (2.3–35.0)	12.1 ± 5.1 (2.5–35.0)	10.7 ± 5.9 (2.3–28.3)
*p* value^b^		0.033*^c^		0.858^c^		0.094^c^		0.193^c^	
Neutrophils, Median [IQR] (Range)	4.40 [2.82–8.15] (0.39–17.85)	4.40 [2.74–8.32] (0.39–17.72)	4.40 [3.26–7.40] (0.98–17.85)	4.40 [2.82–8.32] (0.39–17.85)	4.40 [2.80–7.70] (0.98–13.19)	4.44 [2.76–8.43] (0.39–17.72)	4.36 [3.07–7.40] (0.98–17.85)	4.47 [2.85–8.15] (0.39–17.85)	3.81 [1.41–8.24] (0.98–11.00)
*p* value^b^		0.731^c^		0.912^c^		0.768^c^		0.758^c^	
Lymphocytes, Median [IQR] (Range)	4.30 [2.16–6.62] (0.46–18.70)	4.28 [2.36–6.48] (0.61–16.04)	4.63 [1.90–8.00] (0.46–18.70)	4.32 [2.36–6.60] (0.56–16.04)	2.40 [1.80–8.26] (0.46–18.70)	4.30 [2.39–6.49] (0.61–16.04)	4.20 [1.83–8.00] (0.46–18.70)	4.32 [2.33–6.62] (0.46–16.04)	2.61 [1.52–7.30] (0.89–18.70)
*p* value^b^		0.188^c^		0.917^c^		0.280^c^		0.791^c^	
Platelet count (x10⁹/L), Mean ± SD (Range)	341.9 ± 127.5 (20.3–799.0)	347.0 ± 111.0 (36.0–799.0)	330.9 ± 158.1 (20.3–763.0)	347.1 ± 115.6 (36.0–799.0)	317.8 ± 172.7 (20.3–763.0)	348.2 ± 110.7 (36.0–799.0)	327.2 ± 160.5 (20.3–763.0)	355.0 ± 119.8 (36.0–799.0)	230.4 ± 139.7 (20.3–486.0)
*p* value^b^		0.415^c^		0.214^c^		0.300^c^		<0.001*^c^	
Hematocrit (%), Mean ± SD (Range)	36.3 ± 3.8 (27.0–46.0)	36.5 ± 3.1 (27.2–45.0)	35.9 ± 4.9 (27.0–46.0)	36.5 ± 3.5 (27.0–46.0)	35.4 ± 5.1 (27.0–45.6)	36.3 ± 3.3 (27.0–45.0)	36.2 ± 4.7 (27.0–46.0)	36.4 ± 3.8 (27.0–45.6)	35.3 ± 4.1 (30.0–46.0)
*p* value^b^		0.353^c^		0.204^c^		0.910^c^		0.332^c^	
MCV (fL), Mean ± SD (Range)	79.7 ± 7.5 (47.9–105.5)	78.5 ± 7.2 (47.9–103.0)	82.4 ± 7.4 (65.6–105.5)	78.9 ± 7.0 (47.9–103.0)	83.6 ± 8.5 (67.8–105.5)	78.7 ± 7.5 (47.9–105.5)	82.2 ± 6.8 (65.6–103.0)	79.3 ± 7.5 (47.9–105.5)	83.2 ± 6.7 (67.8–95.8)
*p* value^b^		0.001*^c^		0.001*^c^		0.004*^c^		0.032*^c^	
MCH (pg), Mean ± SD (Range)	26.6 ± 2.3 (19.8–35.4)	26.5 ± 2.2 (19.8–35.4)	26.8 ± 2.3 (21.3–34.9)	26.5 ± 2.2 (19.8–35.4)	27.1 ± 2.5 (23.4–34.9)	26.5 ± 2.3 (19.8–35.4)	26.8 ± 2.1 (21.3–33.6)	26.6 ± 2.3 (19.8–35.4)	26.9 ± 1.8 (23.4–29.4)
*p* value^b^		0.336^c^		0.145^c^		0.572^c^		0.604^c^	
MCHC (g/dL), Mean ± SD (Range)	33.4 ± 1.6 (27.5–40.0)	33.7 ± 1.2 (30.6–36.0)	32.7 ± 2.2 (27.5–40.0)	33.6 ± 1.6 (27.5–40.0)	32.7 ± 1.8 (28.3–35.6)	33.7 ± 1.2 (30.6–36.0)	32.7 ± 2.3 (27.5–40.0)	33.5 ± 1.5 (28.0–40.0)	32.2 ± 2.3 (27.5–35.6)
*p* value^b^		<0.001*^c^		0.011*^c^		<0.001*^c^		0.001*^c^	
RDW%, Mean ± SD (Range)	13.8 ± 1.5 (10.8–20.7)	13.7 ± 1.5 (10.8–20.4)	14.0 ± 1.6 (11.6–20.7)	13.7 ± 1.5 (10.8–20.4)	14.2 ± 1.6 (12.4–20.7)	13.7 ± 1.5 (10.8–20.4)	14.0 ± 1.6 (11.6–20.7)	13.7 ± 1.5 (10.8–20.4)	14.2 ± 1.7 (12.5–20.7)
*p* value^b^		0.124^c^		0.069^c^		0.205^c^		0.285^c^	

^a^ IQR, Interquartile range (25th–75th percentiles); *n*, number; SD, standard deviation.

^b^ * Significant at *p* < 0.05.

^c^ Least-squares adjusted mean difference.

**Table 4:** Radiological imaging and therapeutic regimens in the studied patients according to the investigated outcomes.

Characteristic^a^	All victims	Unfavorable outcomes		Brain insult		Mechanical ventilation		Mortality	
	Overall *N* = 247 (100%)	No *N* = 170 (69%)	Yes *N* = 77 (31%)	No *N* = 211 (85%)	Yes *N* = 36 (15%)	No *N* = 186 (75%)	Yes *N* = 61 (25%)	No *N* = 210 (85%)	Yes *N* = 37 (15%)
I. Radiological findings									
Abnormal CT brain, *n* (%)	25 (10)	0 (0/0)	25 (32/100)	0 (0/0)	25 (69/100)	1 (1/4.0)	24 (39/96)	10 (5/40)	15 (41/60)
*p* value^b^		<0.001*^c^		<0.001*^d^		<0.001*^c^		<0.001*^d^	
Evidence of hypoxic-ischemic encephalopathy, *n* (%)	22 (9)	0 (0/0)	22 (29/100)	0 (0/0)	22 (61/100)	0 (0/0)	22 (36/100)	9 (4/41)	13 (35/59)
*p* value^b^		<0.001*^c^		<0.001*^d^		<0.001*^c^		<0.001*^d^	
Brain edema, *n* (%)	8 (3)	0 (0/0)	8 (10/100)	0 (0/0)	8 (22/100)	1 (1/13)	7 (11/88)	2 (1/25)	6 (16/75)
*p* value^b^		<0.001*^d^		<0.001*^d^		<0.001*^d^		<0.001*^d^	
Abnormal chest x-ray, *n* (%)	145 (59)	93 (55/64)	52 (68/36)	117 (55/81)	28 (78/19)	95 (51/66)	50 (82/34)	128 (61/88)	17 (46/12)
*p* value^b^		0.058^c^		0.012*^c^		<0.001*^c^		0.087^c^	
Hilar & peri-bronchial wall thickening, *n* (%)	87 (35)	65 (38/75)	22 (29/25)	76 (36/87)	11 (31/13)	66 (35/76)	21 (34/24)	80 (38/92)	7 (19/8.0)
*p* value^b^		0.141^c^		0.526^c^		0.881^c^		0.024*^c^	
Field opacities, *n* (%)	38 (15)	16 (9/42)	22 (29/58)	29 (14/76)	9 (25/24)	17 (9/45)	21 (34/55)	33 (16/87)	5 (14/13)
*p* value^b^		<0.001*^c^		0.084^c^		<0.001*^c^		0.732^c^	
Aspiration pneumonia, *n* (%)	18 (7)	9 (5/50)	9 (12/50)	17 (8/94)	1 (3/5.6)	9 (5/50)	9 (15/50)	17 (8/94)	1 (3/5.6)
*p* value^b^		0.073^c^		0.485^d^		0.019*^d^		0.488^d^	
Lung collapse, *n* (%)	8 (3)	4 (2/50)	4 (5/50)	6 (3/75)	2 (6/25)	4 (2/50)	4 (7/50)	6 (3/75)	2 (5/25)
*p* value^b^		0.260^d^		0.329^d^		0.106^d^		0.342^d^	
Pulmonary edema, *n* (%)	6 (2)	0 (0/0)	6 (8/100)	2 (1/33)	4 (11/67)	0 (0/0)	6 (10/100)	4 (2/67)	2 (5/33)
*p* value^b^		<0.001*^d^		0.005*^d^		<0.001*^d^		0.222^d^	
II. Therapeutic regimens									
Vasopressor, *n* (%)	55 (22)	27 (16/49)	28 (36/51)	40 (19/73)	15 (42/27)	32 (17/58)	23 (38/42)	41 (20/75)	14 (38/25)
*p* value^b^		<0.001*^c^		0.002*^c^		<0.001*^c^		0.014*^c^	
Sedation, *n* (%)	49 (20)	1 (1/2.0)	48 (62/98)	22 (10/45)	27 (75/55)	2 (1/4.1)	47 (77/96)	34 (16/69)	15 (41/31)
*p* value^b^		<0.001*^c^		<0.001*^c^		<0.001*^c^		<0.001*^c^	
Intensive antibiotic course, *n* (%)	83 (34)	40 (24/48)	43 (56/52)	58 (27/70)	25 (69/30)	41 (22/49)	42 (69/51)	70 (33/84)	13 (35/16)
*p* value^b^		<0.001*^c^		<0.001*^c^		<0.001*^c^		0.831^c^	
ICU admission, *n* (%)									
No	166 (67)	148 (87/89)	18 (23/11)	163 (77/98)	3 (8/1.8)	162 (87/98)	4 (7/2.4)	150 (71/90)	16 (43/9.6)
Yes	81 (33)	22 (13/27)	59 (77/73)	48 (23/59)	33 (92/41)	24 (13/30)	57 (93/70)	60 (29/74)	21 (57/26)
*p* value^b^		<0.001*^c^		<0.001*^c^		<0.001*^c^		<0.001*^c^	
Length of hospital stay (days), Median [IQR] (Range)	1 [1–2] (1–304)	1 [1–2] (1–5)	2 [1–7] (1–304)	1 [1–2] (1–153)	5.5 [1–18.5] (1–304)	1 [1–2] (1–5)	3 [1–10] (1–304)	1 [1–2] (1–304)	2 [1–6] (1–153)
*p* value^b^		<0.001*^e^		<0.001*^e^		<0.001*^e^		0.053^e^	
Length of ICU stay (days), Median [IQR] (Range)	5 [2–12] (1–69)	1 [1–2] (1–2)	6 [4–19] (1–69)	2 [1–4] (1–34)	12 [6–27] (2–69)	1 [1–1.5] (1–2)	6 [4–19] (1–69)	3 [1–13] (1–69)	6 [5–12] (1–23)
*p* value^b^		<0.001*^e^		<0.001*^e^		<0.001*^e^		0.193^e^	

Percents are expressed as column/row percentages.

^a^ IQR, interquartile range (25th–75th percentiles); n: Number; SD: Standard deviation

^b^ * Significant at *p* < 0.05.

^c^ Pearson's chi-square test.

^d^ Fisher's exact test.

^e^ Wilcoxon rank sum test.

**Figure 1:**
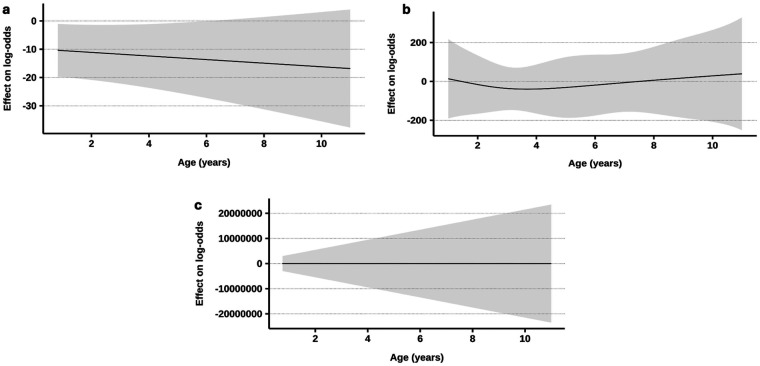
Relationships between age (in years) and log-odds ratios for three outcome models (a. model for predicting brain insults; b. model for predicting respiratory failure with a need for mechanical ventilation; c. model for predicting mortality). In all the models, the smooth age terms were statistically nonsignificant, indicating that age does not serve as a meaningful predictor or confounder.

Table 5 shows the three models for predicting individual unfavorable outcomes. The model for the prediction of brain insults exhibited strong performance (Nagelkerke R²=0.920), identifying the admission GCS score as the only significant predictor. Higher GCS scores reflect better neurological function, thus reducing the likelihood of brain insults. A lower GCS score at admission [odds ratio (OR) = 0.318], lower pH (OR = 0.000), lower MCH (OR = 0.209), and lower RDW (OR = 0.052) were identified as risk factors that could significantly predict the need for MV. The third model predicting mortality revealed that the lower the bicarbonate level was, the greater the risk of mortality (OR = 0.463). An OR of 0.655 suggests that a higher chloride level is associated with a decreased risk of mortality. Patients with higher RDWs and higher sodium levels at admission had 3.143- and 2.262-fold greater odds of mortality, respectively. After CPR and loss of consciousness were included as confounding factors in the proposed models, the changes in predictors of brain insults were minimal (<3% for most variables; delay ≈19%). Moreover, in the MV prediction model, the coefficients for delay, pH, and RDW changed substantially (30%–82%), suggesting partial confounding by CPR and loss of consciousness, whereas Cl, MCH, and MCHC remained largely unaffected. Nevertheless, all the predictors lost their significance. Additionally, the inclusion of CPR and loss of consciousness changed the coefficients for HCO₃, Na, Cl, and RDW by <10%, indicating that these laboratory variables are independent predictors of mortality. The potential contribution of age to the investigated outcomes was assessed. Across all three outcomes, modeling age as a continuous and flexible smooth term showed no clear linear or nonlinear association with the endpoints and only small (3%–36%) changes in other coefficients (Supplementary Material). Figure 1. The smooth terms for age in the GAMs were consistently nonsignificant and did not reveal any discernible patterns, reinforcing the notion that age acts neither as a critical predictor nor as a significant confounder in this analysis.

**Table 5:** Multivariate models for predicting unfavorable outcomes in the studied patients with and without adjustment for severity (cardiopulmonary resuscitation and loss of consciousness).

Models without adjustment			Models with adjustment		
I. Models predicting brain insults					
$\log\;\mathrm{odd}s_{\mathrm{brain}\;\mathrm{insult}}=66.135+0.33(\mathrm{Delay})-2.22(\mathrm{GC}S_{\mathrm{admission}})+0.533(\mathrm{HC}O_{3})$ −0.361(Na)+0.305(RBS)−1.144(RDW)			$\log\;\mathrm{odd}s_{\mathrm{brain}\;\mathrm{insult}}=64.845+0.392(\mathrm{Delay})-2.249(\mathrm{GC}S_{\mathrm{admission}})+0.536(\mathrm{HC}O_{3})$ −0.367(Na)+0.308(Bloofglucoselevel)−1.175(RDW) +0.544(CPRdone)+2.154(LOCbeforeadmission)		
Characteristic	Adjusted OR (95% CI)	*p* value^a^	Characteristic	Adjusted OR (95% CI)	*p* value^a^
Intercept		0.050*	Intercept		0.048*
Delay time (hr)	1.391 (0.868–2.406)	0.185	Delay time (hr)	1.480 (0.870–3.400)	0.276
GCS on admission	0.109 (0.006–0.369)	0.019*	GCS on admission	0.106 (0.006–0.368)	0.021*
HCO₃ (mEq/L)	1.703 (1.133–4.045)	0.066	HCO₃ (mEq/L)	1.710 (1.130–4.184)	0.070
Na (mmol/L)	0.697 (0.407–0.930)	0.054	Na (mmol/L)	0.693 (0.397–0.929)	0.056
Blood glucose (mmol/L)	1.356 (1.057–2.194)	0.063	Blood glucose (mmol/L)	1.361 (1.058–2.241)	0.066
RDW	0.318 (0.035–1.355)	0.179	RDW	0.309 (0.032–1.344)	0.176
			CPR done	1.723 (0.002–2,266.395)	0.888
			LOC before admission	8.618 (0.000–276,081.093)	0.806
Null deviance/deviance = 126/16.1, Nagelkerke's *R*^2^ = 0.920, Likelihood ratio test = <0.001, Hosmer‒Lemeshow test = 1.000, AIC = 30.1			Null deviance/deviance = 126/16.1, Nagelkerke's *R*^2^ = 0.920, Likelihood ratio test = <0.001, Hosmer‒Lemeshow test = 1.000, AIC = 34.0		
II. Models predicting respiratory failure with a need for mechanical ventilation					
$\log\;\mathrm{odd}s_{\mathrm{mechanical}\;\mathrm{ventilation}}$ $=200.146+0.126(\mathrm{Delay})-1.144(\mathrm{GC}S_{\mathrm{admission}})$ −28.94(pH)+0.809(Cl)−1.564(MCH) +0.472(MCHC)−2.962(RDW)			$\log\;\mathrm{odd}s_{\mathrm{mechanical}\;\mathrm{ventilation}}=316.099+0.022(\mathrm{Delay})-1.438(\mathrm{GC}S_{\mathrm{admission}})$ −44.627(pH)+0.690(Cl)−1.278(MCH)+0.422(MCHC)−2.069(RDW)−3.875(CPRdone)) −2.634(Lostconsciousnessbeforeadmission		
Characteristic	Adjusted OR (95% CI)	*p* value^a^	Characteristic	Adjusted OR (95% CI)	*p* value^a^
Intercept		0.036*	Intercept		0.085
Delay time (hr)	1.134 (0.349–1.216)	0.835	Delay time (hr)	1.022 (0.185–1.175)	0.980
GCS on admission	0.318 (0.091–0.617)	0.011*	GCS on admission	0.237 (0.020–0.564)	0.036*
pH	Per 0.01 pH: 0.749 (0.483–0.914)	0.043*	pH	Per 0.01 pH: 0.640 (0.243–0.878)	0.090
Cl (mmol/L)	2.245 (1.080–7.507)	0.089	Cl (mmol/L)	1.993 (0.929–7.811)	0.180
MCH (pg)	0.209 (0.026–0.689)	0.044*	MCH (pg)	0.279 (0.025–1.160)	0.165
MCHC (g/dL)	1.604 (0.866–5.801)	0.215	MCHC (g/dL)	1.526 (0.616–7.523)	0.435
RDW	0.052 (0.002–0.432)	0.026*	RDW	0.126 (0.004–0.968)	0.108
			CPR done	0.021 (0.000–4.415)	0.251
			LOC before admission	0.072 (0.000–520.566)	0.573
Null deviance/deviance = 109/15.1, Nagelkerke's *R*^2^ = 0.923, Likelihood ratio test = <0.001, Hosmer‒Lemeshow test = 1.000, AIC = 31.1			Null deviance/deviance = 109/12.2, Nagelkerke's *R*^2^ = 0.923, Likelihood ratio test = <0.001, Hosmer‒Lemeshow test = 1.000, AIC = 32.2		
III. Models predicting the mortality					
$\log\;\mathrm{odd}s_{\mathrm{Mortality}}=-72.659-0.771(\mathrm{HC}O_{3})+0.816(\mathrm{Na})-0.423(\mathrm{Cl})+1.145(\mathrm{RDW})$ ^b^			$\log\;\mathrm{odd}s_{\mathrm{Mortality}}=-51.255\;-0.514(\mathrm{HC}O_{3})+\;0.589(\mathrm{Na})-0.327(\mathrm{Cl})+0.769(\mathrm{RDW})\;-1.367(\mathrm{CPR}\;\mathrm{done})$ +2.643(LOCbeforeadmission)		
Characteristic	Adjusted OR (95% CI)	*p* value^a^	Characteristic	Adjusted OR (95% CI)	*p* value^a^
Intercept		0.001*	Intercept		<0.001*
HCO₃ (mEq/L)	0.463 (0.253–0.658)	0.001*	HCO₃ (mEq/L)	0.598 (0.451–0.793)	<0.001*
Na (mmol/L)	2.262 (1.514–4.386)	0.001*	Na (mmol/L)	1.802 (1.303–2.493)	<0.001*
Cl (mmol/L)	0.655 (0.440–0.861)	0.009*	Cl (mmol/L)	0.721 (0.567–0.918)	0.008*
RDW	3.143 (1.618–9.062)	0.005*	RDW	2.158 (1.210–3.849)	0.009*
			CPR done	0.255 (0.010–6.227)	0.402
			LOC before admission	14.057 (0.199–992.775)	0.224
Null deviance/deviance = 74.4/22.1, Nagelkerke's R^2^ = 0.774, Likelihood ratio test = <0.001, Hosmer‒Lemeshow test = 1.000, AIC = 32.1			Null deviance/deviance = 74.4/22.0, Nagelkerke's *R*^2^ = 0.775, Likelihood ratio test = <0.001, Hosmer‒Lemeshow test=0.989, AIC = 337.17		

AIC, Akaike information criterion; CI, confidence interval; CPR, cardiopulmonary resuscitation; LOC, loss of consciousness; OR, odds ratio.

^a^ *p < 0.05.

^b^ Firth's bias-reduction method was used owing to the quasiseparation observed after including the loss of consciousness variable.

## 4 Discussion

This study, a 10-year retrospective analysis conducted in a tertiary care center, aimed to refine the predictive criteria for poor prognosis, specifically mortality and other life-threatening outcomes in pediatric near-drowning patients. The observed mortality rate of 15% is consistent with rates reported in previous studies (7, 20). Previous studies estimated that survivors constitute two or three fatalities (21, 22). The epidemiological patterns observed in this cohort align with established trends in pediatric near-drowning. The male predominance and peak incidence during the summer season, with most incidents occurring at night, were reported earlier (7, 23). Notably, swimming pools, which are prevalent amenities in Saudi Arabia, accounted for 85% of the cases, which is consistent with previous studies (7, 23, 24). These findings contrast with previous reports where natural water sources, rivers, and canals were more common settings of drowning in some Western countries (21, 22, 25, 26). This difference underscores the importance of targeted, localized prevention strategies focused on pool safety, particularly during peak hours and seasons.

The unintentional nature of drowning was consistent with established patterns of pediatric drowning (23, 24). Drowning is a rare pattern of suicidal death, accounting for less than 1% of all suicides (27). The potential for intentional drowning increases with increasing age. Ağrıtmıs et al. considered drowning the third cause of suicide in childhood and adolescence, following firearm injuries and hanging (28). Compared with other methods of self-harm, intentional drowning is associated with an increased risk of death (29). Careful investigation and documentation of the circumstances of the incident, especially in older children, are mandatory. Transitioning children's swimming locations from natural water sources to more monitored environments, such as pools and beaches with trained lifeguards or bystanders skilled in CPR, improves survival rates (30).

With respect to transport, approximately 70% of the cases involved transport via private vehicles, which was associated with favorable outcomes. Conversely, helicopter transport was significantly associated with severe outcomes. Lousx et al. reported contradictory findings, where transport by ambulance was the most common mode of transport. Moreover, helicopter transport was significantly associated with severity at admission (7). This finding reflects the severity of the initial injury necessitating rapid, specialized transport rather than the transport itself causing adverse outcomes. The time to emergency service arrival has emerged as a critical determinant of mortality. Cardiac arrest on/during admission was significantly associated with unfavorable outcomes, which was reported earlier (7). Suominen et al. considered the need for advanced life supportive measures at the scene a factor determining survival with minimal neurological involvement (6). Although CPR is crucial for survival, its initiation often indicates a more severe initial event. In this study, it was difficult to confirm whether only patients with cardiac arrest had received CPR on the scene, which might explain the observed association between CPR and severe outcomes.

Since the brain and lungs are the main organs affected by drowning, brain injury and cardiac arrest are the leading causes of long-term complications (31–33). While there is a global consensus on the necessity of aggressive respiratory management, preventing brain damage is challenging (34). Our findings are consistent with those of previous studies indicating that a lower body temperature during drowning is a poor prognostic sign, reflecting fast cooling or extended submersion (22, 35–37). Increased cardiac work, increased oxygen demand and a catecholamine crisis, coupled with cold-water swimming failure, usually lead to death, particularly in patients with heart problems, such as long QT syndrome (LQTS) (34). Therefore, intubation should be performed for drowned victims with inadequate ventilation (34). Other factors support the protective effect of hypothermia. Owing to their relatively high surface area‒to‒weight ratio, the rapid decrease in body temperature in children is a key factor that allows them to survive prolonged periods of hypoxia, often without lasting brain damage (38). Hypothermia slows metabolism and decreases oxygen demand, exerting a protective effect on the brain (21, 22, 34, 39).

The most significant contribution of this study is the identification of objective laboratory markers that serve as potential independent predictors of poor outcomes, complementing the established clinical assessment tools. This study identified low MCH and RDW as predictors for MV, whereas increased RDW could predict mortality. Indeed, the associations between the MCH and RDW and the need for MV reflect the severity (need for CPR and loss of consciousness on admission) rather than a concrete correlation. The body's oxygen-carrying capacity becomes critically important in drowning, which often leads to hypoxia and ischemia. Reduced oxygen delivery can exacerbate the effects of the initial hypoxic insult from drowning, leading to more severe tissue damage and organ dysfunction, particularly in the respiratory system. In other critical conditions, a low MCH is associated with poorer prognosis and reflects increased severity of illness (40). However, the observed association between mortality and increased RDWs is attributed to associated inflammation and oxidative stress, similar to the reported associations between high RDWs and death in various critical illnesses (41–43).

In patients with unfavorable outcomes, the significantly reduced pH and bicarbonate levels, hyperglycemia and electrolyte imbalances further illustrate the widespread physiological disruption in near-drowning. In accordance with these findings, pH > 7 (44, 45) and a blood glucose level < 11.2 mmol (46, 47) were found to be significant predictors of a favorable outcome in drowning patients in previous studies. Son et al. considered a pH < 7.2, a bicarbonate level < 15 mEq/L and a blood glucose level >200 mg/dL to be significant predictors of ischemic encephalopathy and death (18). These metabolic disturbances might indicate prolonged submersion times (21, 33, 37, 48–52). The observed associations between electrolyte imbalances, including hypernatremia, and mortality are consistent with the findings of previous studies (18, 53). These findings might reflect renal impairments, as indicated by increased creatinine levels, which can further disturb electrolytes (54).

The observed findings substantiated previous findings that the GCS score is a negative predictor of unfavorable outcomes. Consistently, low GCS scores on admission were shown to predict death and other unfavorable outcomes in previous studies (18, 34, 45, 47, 55). Coma size and fixed dilated pupils are reliable predictors of mortality and permanent brain damage (56, 57). The reactivity of the pupil on admission could discriminate between survivors and nonsurvivors but cannot predict those who are in a vegetative state (58). Regained consciousness, spontaneous movements, and intact brain stem functions after submersion are predictors of complete recovery (58, 59). Indeed, the pupillary reflex is intact even in patients under sedation, making it a good sign for monitoring deteriorated patients on ventilation (21).

The integration of clinical and laboratory predictors demands attention from clinicians and policymakers. Clear, evidence-based prognosis supports physicians in communicating with families and provides necessary legal documentation in cases of suspected brain death or futility of care. Repeated neurological examinations following discharge until adulthood are necessary to avoid adverse neurological consequences (6). The observed association between the absence of CPR at the scene and increased mortality underscores the legal and ethical imperative for widespread bystander CPR training (60).

It is important to comprehensively investigate the circumstances of drowning. The possibility of intentional drowning in adolescents or child neglect/abuse in younger children requires a thorough medicolegal approach. The medical team's documentation, including the objective predictors identified here, serves as crucial evidence for child protective services and legal authorities (61). Given the observed unintentional nature of all investigated drowning incidents, the potential for underlying cardiac conditions as a cause of drowning should be excluded. The occurrence of near-drowning, particularly in the absence of clear environmental factors, should prompt clinicians to consider underlying cardiac disorders. Cardiac arrhythmias may be an important consideration for near-drowning survivors and their families when a plausible explanation is lacking (62). Long QT syndrome is a genetic disorder characterized by prolonged ventricular repolarization that is associated with syncope and sudden death. Electrocardiographic (ECG) screening programs and genetic testing for first-degree family members are associated with reduced mortality and other associated life-threatening events (63).

The observed findings provide an evidence base for public health policy. The high drowning incidence in swimming pools, especially at night, supports the need for stricter regulations on pool fencing, supervision, and alarms, which can be legally mandated to reduce preventable deaths. It may be worth attempting to plan and implement educational public measures, focusing on preventive measures and bystander intervention, which might reduce the incidence of mortality and other adverse outcomes (7).

## 5 Strengths and limitations

One strength of this study is that it was conducted in multiple centers, which allows for the generalizability of the findings obtained for local and regional decision-making. Nevertheless, an inherent limitation of the retrospective nature of the current study is the scarcity of some data concerning exposure circumstances, including the presence of a supervising attendee and the duration of downtime submersion. Another limitation of this retrospective study is the lack of related autopsy data. Indeed, the ICD used by most tertiary care centers might not precisely reflect the actual incidence of drowning. Likewise, some patients who drown with short downtime periods may regain consciousness after resuscitation without admission. On the other hand, children admitted with long submersion times who suffer from hypoxic brain injury might pass away after discharge because of pneumonia or other complications but not death due to drowning. We acknowledge the lack of long-term follow-up and recommend that future studies address this gap. We also recommend conducting prospective autopsy-based studies to provide further insight into underlying cardiac or other pathologies.

## 6 Conclusions

This study underscores the factors associated with unfavorable outcomes in pediatric near-drowning incidents. The establishment of a standardized clinical protocol for assessing near-drowning victims is urgently needed. This initial assessment should incorporate the reported readily available laboratory markers, including bicarbonate and pH levels, serum electrolytes, and CBC indices, namely, the RDW. Given the significant proportion of near-drowning events that occur in swimming pools, we recommend implementing community-based educational initiatives aimed at caregivers about the dangers of drowning in pool environments and promoting supervision practices during high-risk periods, such as the summer months and nighttime. The observed association between the delay in obtaining emergency services and the development of unfavorable outcomes highlights the need to develop targeted training for emergency responders to prioritize rapid transport to medical facilities and minimize delays in such situations.

## Data Availability

The raw data supporting the conclusions of this article will be made available by the authors, without undue reservation.
