# Supplementary material for: Pediatric near-drownings: clinical insights and prediction of life-threatening events
Source: Front Pediatr. 2026 Jan 12;13:1700437. doi: 10.3389/fped.2025.1700437 (PMC12832822; doi:10.3389/fped.2025.1700437)
Supplement: Supplementary file 1 [file Table1.docx]

**Supplementary. Potential contribution of age to the proposed models predicting the investigated outcomes**

| **Characteristic** | **No age** | | | **with age** | | |
| --- | --- | --- | --- | --- | --- | --- |
|  | **B** | **95% CI** | **p-value** | **B** | **95% CI** | **p-value** |
| 1. **Models predicting brain insults** | | | | | | |
| **Delay time from drowning till emergency treatment (hr)** | 0.39 | -0.14 to 1.2 | 0.276 | 0.41 | -0.12 to 1.4 | 0.300 |
| **GCS on admission** | -2.2 | -5.2 to -1.0 | 0.021 | -2.3 | -5.6 to -1.0 | 0.027 |
| **HCO₃ (mEq/L)** | 0.54 | 0.12 to 1.4 | 0.070 | 0.59 | 0.16 to 1.6 | 0.052 |
| **Na (mmol/L)** | -0.37 | -0.92 to -0.07 | 0.056 | -0.39 | -1.0 to -0.09 | 0.051 |
| **Random blood glucose (mmol/L)** | 0.31 | 0.06 to 0.81 | 0.066 | 0.32 | 0.07 to 0.78 | 0.046 |
| **RDW** | -1.2 | -3.5 to 0.30 | 0.176 | -1.4 | -3.9 to 0.18 | 0.140 |
| **CPR done** | 0.54 | -6.4 to 7.7 | 0.888 | 0.81 | -7.2 to 9.2 | 0.900 |
| **LOC before admission** | 2.2 | -18 to 13 | 0.806 | 1.5 | -21 to 12 | 0.900 |
| **Age (Years)** | --- | --- | --- | -0.63 | -3.2 to 0.51 | 0.500 |
| 1. **Models predicting respiratory failure with a need for mechanical ventilation** | | | | | | |
| **Delay time from drowning till emergency treatment (hr)** | 0.02 | -  -1.169 to 0.162 | 0.980 | 0.07 | -0.93 to 1.06 | 0.900 |
| **GCS on admission** | -1.4 | -3.9 to -0.57 | 0.036 | -1.4 | -4.2 to -0.56 | 0.044 |
| **pH** | -45 | -141 to -13 | 0.090 | -43 | -136 to -12 | 0.082 |
| **Cl (mmol/L)** | 0.69 | -0.07 to 2.1 | 0.180 | 0.84 | -0.01 to 2.6 | 0.140 |
| **MCH (pg)** | -1.3 | -3.7 to 0.15 | 0.165 | -1.5 | -4.8 to 0.03 | 0.140 |
| **MCHC (g/dL)** | 0.42 | -0.48 to 2.0 | 0.435 | 0.25 | -0.84 to 1.8 | 0.600 |
| **RDW** | -2.1 | -5.5 to -0.03 | 0.108 | -1.6 | -5.3 to 0.63 | 0.200 |
| **CPR done** | -3.9 | -14 to 1.5 | 0.251 | -3.7 | -15 to 1.7 | 0.300 |
| **LOC before admission** | -2.6 | -15 to 6.3 | 0.573 | -2.0 | -16 to 8.6 | 0.700 |
| **Age (Years)** | --- | --- | --- | 0.62 | -0.59 to 2.8 | 0.400 |
| 1. **Models predicting the mortality** | | | | | | |
| **HCO₃ (mEq/L)** | -0.51 | -0.80 to -0.23 | <0.001 | -0.61 | -1.0 to -0.21 | 0.003 |
| **Na (mmol/L)** | 0.59 | 0.26 to 0.91 | <0.001 | 0.78 | 0.29 to 1.3 | 0.002 |
| **Cl (mmol/L)** | -0.33 | -0.57 to -0.09 | 0.008 | -0.45 | -0.78 to -0.12 | 0.008 |
| **RDW** | 0.77 | 0.19 to 1.3 | 0.009 | 0.86 | 0.02 to 1.7 | 0.044 |
| **CPR done** | -1.4 | -4.6 to 1.8 | 0.402 | -2.2 | -6.3 to 1.8 | 0.300 |
| **LOC before admission** | 2.6 | -1.6 to 6.9 | 0.224 | 1.7 | -2.2 to 5.6 | 0.400 |
| **Age (Years)** | --- | --- | --- | -2.9 | -5.6 to -0.09 | 0.043 |

CI: confidence interval; CPR: cardiopulmonary resuscitation; LOC: loss of consciousness; *a:* *p<0.05
